# A Direct Coarray Interpolation Approach for Direction Finding

**DOI:** 10.3390/s17092149

**Published:** 2017-09-19

**Authors:** Tao Chen, Muran Guo, Limin Guo

**Affiliations:** 1 College of Information and Communication Engineering, Harbin Engineering University, No. 145 Nantong Street, Harbin 150001, China; chentao@hrbeu.edu.cn (T.C.); guomuran@hrbeu.edu.cn (M.G.); 2 Depaprtment of Electrical and Computer Engineering, Temple University, Philadelphia, PA 19122, USA

**Keywords:** coarray interpolation, DOA estimation, MUSIC, nuclear norm, sparse array

## Abstract

Sparse arrays have gained considerable attention in recent years because they can resolve more sources than the number of sensors. The coprime array can resolve O(MN) sources with only O(M+N) sensors, and is a popular sparse array structure due to its closed-form expressions for array configuration and the reduction of the mutual coupling effect. However, because of the existence of holes in its coarray, the performance of subspace-based direction of arrival (DOA) estimation algorithms such as MUSIC and ESPRIT is limited. Several coarray interpolation approaches have been proposed to address this issue. In this paper, a novel DOA estimation approach via direct coarray interpolation is proposed. By using the direct coarray interpolation, the reshaping and spatial smoothing operations in coarray-based DOA estimation are not needed. Compared with existing approaches, the proposed approach can achieve a better accuracy with lower complexity. In addition, an improved angular resolution capability is obtained by using the proposed approach. Numerical simulations are conducted to validate the effectiveness of the proposed approach.

## 1. Introduction

Array signal processing, including beamforming and direction of arrival (DOA) estimation, is of great interest [1,2,3,4,5,6,7,8,9]. Compared with classical uniform linear arrays (ULAs), many sparse array structures (e.g., coprime array [10,11,12] and nested array [13]) have been proposed in the last decades to achieve a higher number of degrees of freedom (DOFs) and improve the array processing capability. By using a nested array, $O(N^{2})$ uncorrelated sources can be resolved by O(N) antennas. However, the nested array structure requires that the inter-element spacing of one subarray is half wavelength, thus leading to heavy mutual coupling effects. To address this issue, the coprime array was proposed in [10] and further developed in [11,12]. Especially, the mutual coupling effects are significantly reduced by using coprime arrays with displaced subarrays (CADiS) [12]. However, one shortcoming of coprime arrays is that there are holes in the resultant difference coarray (i.e., not all the lags are consecutive). As a result, the number of DOFs is reduced for subspace-based algorithms, such as multiple signal classification (MUSIC) [14] and estimation of signal parameters via rotational invariance techniques (ESPRIT) [15], since the spatial smoothing (SS) technique can only use the consecutive lags.

MUSIC is a classical super-resolution subspace-based DOA estimation algorithm that has been widely used. For coarray-based MUSIC [16], a reshaping operation is first applied to the covariance matrix to obtain the equivalent received data vector of the coarray. Since the equivalent received data vector is extracted directly from the covariance matrix of the received data, the rank of the covariance matrix of coarray is one, thus making this problem similar with operating coherent sources. As such, we need to recover the rank of the covariance matrix of the coarray by using the SS technique. Then, the rank-recovered covariance matrix can be utilized to perform the MUSIC algorithm. Note that the conventional MUSIC needs a spatial grid to estimate the DOAs. More accurate estimation can be acquired by exploiting a denser grid, thus leading to a trade-off between the computational complexity and the estimation performance.

As mentioned above, the lags out of the consecutive range cannot be utilized for SS due to the existence of holes in the coarray when the coprime array is used. Several coarray interpolation approaches have been proposed to overcome this problem [17,18,19]. In [18], a simple approach to fill the holes was proposed by optimizing a nuclear norm minimization problem. In [19], a denoising operation was added to decrease the randomness caused by the finite number of snapshots, thus achieving a more accurate estimation. However, the computational complexity is a problem, since two convex problems are optimized. In this paper, a direct coarray interpolation approach is proposed. Only one nuclear norm optimization problem is optimized in the proposed approach. In addition, the covariance matrix of the coarray is directly recovered from the covariance matrix of received data without the reshaping operation. Furthermore, the rank of the covariance matrix is recovered, which means that MUSIC can be readily performed to estimate the DOAs. Note that the noise is also suppressed via the proposed nuclear norm minimization problem. As such, an accurate DOA estimation can be obtained with lower complexity, and the angular resolution is improved through the proposed approach. In addition, it is worth noting that the proposed approach is focused on obtaining the interpolated and denoised covariance matrix. So, MUSIC is not the only DOA estimation algorithm that can be used. Actually, all the subspace-based algorithms can be perform to retrieve the DOAs, such as the recently proposed coarray ESPRIT [20]. Numerical simulations validate the effectiveness of the proposed approach.

The rest of this paper is organized as follows. In Section 2, we briefly introduce the signal model for DOA estimation based on sparse arrays and the principle of coarray-based MUSIC. Then, the proposed approach is derived in Section 3. Simulation results are shown in Section 4 to examine the performance of the proposed approach. In Section 5, we give the conclusion of this paper.

Notations: we use the lower-case letter (*a*), lower-case bold letter (a), and upper-case bold letter (A) to represent the scalars, vectors, and matrices, respectively. The superscripts *, *T*, and *H* denote the complex conjugate, the transpose, and the complex conjugate transpose. Let vec(·) and E(·) represent the vectorization and expectation operation. The diagonal matrix whose diagonal entries are given in a is expressed by diag(a). $j=\sqrt{-1}$ is the complex symbol, and $I_{L}$ is the L×L identity matrix. We use the triangle bracket ${\langle x_{S}\rangle }_{n}$ to represent the value corresponding to the support n∈S. For example, let $x_{S}=\left\{2,3,4\right\}$ and S={−1,0,1}. Then, we have ${\langle x_{S}\rangle }_{-1}=2$, ${\langle x_{S}\rangle }_{0}=3$ and ${\langle x_{S}\rangle }_{1}=4$.

## 2. Preliminaries

In this section, we first introduce the signal model of DOA estimation using sparse arrays. Then, the methods of coarray-based MUSIC are demonstrated.

### 2.1. Signal Model

Assume that a sensor array is illuminated by *Q* far-field narrowband and uncorrelated sources with DOAs $\theta_{q}$ satisfying $-\pi /2\leq \theta_{q}\leq \pi /2$ for q=1,2,…,Q. In addition, the sensors are located at $\left\{d_{1}d_{0},\ldots ,d_{L}d_{0}\right\}$, where $\left\{d_{1},\ldots ,d_{L}\right\}$ is an integer set and $d_{0}=\lambda /2$ is the unit inter-element spacing with λ denoting the wavelength. For simplicity, set $d_{1}=0$. Let *M* and *N* be a pair of co-prime integers. Without loss of generality, assume that M<N. Denote $\left\{d_{1},\ldots ,d_{L}\right\}$ by S. For coprime arrays, S is given by

(1) S={0,M,…,(N−1)M}∪{0,N,…,(2M−1)N}.

Then, the received data vector is expressed as

(2) $$\begin{array}{c} x(t)=As(t)+n(t),\;t=1,\;2,\;\ldots ,\;T, \end{array}$$

where *T* is the number of snapshots, and n(t) is the additive white Gaussian noise with mean zero and covariance matrix $\sigma_{n}^{2}I_{L}$. $A\;=\;\left[a\left(\theta_{1}\right),\;\ldots ,\;a\left(\theta_{Q}\right)\right]$ is the manifold matrix, and $a(\theta_{q})$ is the steering vector of the *q*th source, expressed as

(3) $$\begin{array}{cc} a(\theta_{q})= & {\left[e^{j2\pi d_{1}d_{0}\sin\left(\theta_{q}\right)/\lambda },\ldots ,e^{j2\pi d_{L}d_{0}\sin\left(\theta_{q}\right)/\lambda }\right]}^{T}. \end{array}$$

Theoretically, the covariance matrix of x(t) is expressed as

(4) $$R_{S}=E\left[x\left(t\right)x^{H}\left(t\right)\right]=AR_{ss}A^{H}+\sigma_{n}^{2}I_{L},$$

where $R_{ss}=\mathrm{diag}\left[\sigma_{1}^{2},\;\ldots ,\;\sigma_{Q}^{2}\right]$ is the covariance matrix of the sources with $\sigma_{q}^{2}$ denoting the power of the *q*th source.

Let V be the shortest ULA that contains S; i.e., the ULA distributed from $d_{1}$ to $d_{L}$. Thus, $R_{V}$, the covariance matrix of V, is given by

(5) $$R_{V}=A_{V}R_{ss}A_{V}^{H}+\sigma_{n}^{2}I_{d_{L}+1},$$

where $A_{V}$ is the manifold matrix corresponding to V. Then, the relationship between $R_{S}$ and $R_{V}$ can be expressed as

(6) $$R_{S}=Q^{T}R_{V}Q,$$

where $Q=\left[e_{d_{1}+1},\ldots ,e_{d_{L}+1}\right]$ is the selection matrix. $e_{i}$ is a column vector in which the *i*th entry is 1 while other entries are 0.

In practice, the exact covariance matrix $R_{S}$ in Equation (4) cannot be obtained due to the finite number of snapshots. Thus, the sample covariance matrix ${\tilde{R}}_{S}$, which is expressed as

(7) $${\tilde{R}}_{S}=\frac{1}{T}\sum_{t=1}^{T}x\left(t\right)x^{H}\left(t\right),$$

is used to approximate the $R_{S}$. Then, ${\tilde{R}}_{S}$ can be rewritten as

(8) $${\tilde{R}}_{S}=AR_{ss}A^{H}+\tilde{E},$$

where $\tilde{E}$ is the error term which consists of two parts, $\sigma_{n}^{2}I_{L}$ and the error introduced due to a finite number of snapshots.

### 2.2. Coarray-Based MUSIC

The (i,k)-th entry of $R_{S}$ is expressed as

(9) $$R_{S(i,k)}=\sum_{q=1}^{Q}\sigma_{q}^{2}e^{j2\pi \left(d_{i}-d_{k}\right)\omega_{q}}+\delta_{i,k}\sigma_{n}^{2},$$

where $\omega_{q}=d_{0}\sin\theta_{q}/\lambda$ is the normalized DOA of the *q*-th source. $\delta_{i,k}$ equals 1 when i=k, otherwise, $\delta_{i,k}$ equals 0. Here, we define the difference coarray as

(10) $$D=\{d_{i}-d_{k}|i,k=1,2,\ldots ,L\}.$$

As such, (9) can be considered as the received signal of difference coarray D. Note that the sources for coarray (i.e., $\sigma_{q}^{2}$) are the power of the sources impinging on the physical array. However, there are many overlapped lags in coarray. To perform MUSIC, we should first extract the unique lags in D. The definition of the selection matrix can be found in [16,21]. The signal model after selecting the unique lags is expressed as

(11) $$x_{D}=A_{D}p+\sigma_{n}^{2}e_{0},$$

where $A_{D}=\left[a_{D}\left(\theta_{1}\right),\ldots ,a_{D}\left(\theta_{Q}\right)\right]$ is the manifold matrix of coarray and $a_{D}\left(\theta_{q}\right)$ is the steering vector of coarray with repect to the *q*-th target. $p={\left[\sigma_{1}^{2},\sigma_{2}^{2},\ldots ,\sigma_{Q}^{2}\right]}^{T}$ is a column vector whose elements are the power of sources and $e_{0}$ is a column vector satisfying ${\langle e_{0}\rangle }_{m}=\delta_{m,0}$. Note that only one snapshot of $x_{D}$ is obtained here, thus making this problem similar with operating the coherent sources. As such, SS technique is required to recover the rank of $R_{D}$, the covariance matrix of coarray. Since the lags for the coprime array are not all consecutive, denote [−ξ,−ξ+1,…,ξ−1,ξ] as the range of the consecutive lags in the corresponding coarray. Then, the principle of SS to recover the rank is [13]:

(12) $$R_{e1}=\frac{1}{\xi +1}\sum_{l=0}^{\xi }z_{l}z_{l}^{H},$$

where $z_{l}$ is a subset of $x_{D}$ which is defined as $z_{l}={\left[{\langle x_{D}\rangle }_{-\xi +l},\ldots ,{\langle x_{D}\rangle }_{l}\right]}^{T}$. Recently, a direct augmented approach (DAA) was proposed to recover the rank [22]. By using DAA, the covariance matrix can be recovered via a simple reshaping operation as

(13) $$R_{e2}=\left[z_{\xi },z_{\xi -1},\ldots ,z_{0}\right].$$

As analyzed in [16], although the SS technique is a quadratic operation while DAA is a linear operation, the two methods have the same DOA estimation performance. After restoring the rank, MUSIC can be used to estimate the DOAs. For simplicity, we use $R_{e1}$ in the following demonstrations unless otherwise specified. First, perform eigendecomposition on $R_{e1}$

(14) $$R_{e1}=\left[\begin{array}{cc} U_{S} & U_{N} \end{array}\right]\Sigma {\left[\begin{array}{cc} U_{S} & U_{N} \end{array}\right]}^{H},$$

where $U_{S}$ is the signal subspace whose columns are the eigenvectors that correspond to the *Q* largest eigenvalues. Similarly, $U_{N}$ is the noise subspace consisting of the remaining eigenvectors. Σ is the eigenvalue matrix whose diagonal entries are the eigenvalues in descending order while other entries are zeros. The main method of MUSIC is to utilize the orthogonality between the signal subspace and the noise subspace. Since the signal subspace is equivalent to the space spanned by the steering vectors, the steering vectors are also orthogonal to the noise subspace; i.e., $a^{H}\left(\theta_{q}\right)U_{N}=0$. By dividing the interest region into a dense grid, we can estimate the DOA by finding the θ that minimizes $a^{H}\left(\theta \right)U_{N}U_{N}^{H}a\left(\theta \right)$. As such, the spatial spectrum of MUSIC is defined as

(15) $$P_{MUSIC}=\frac{1}{a^{H}\left(\theta \right)U_{N}U_{N}^{H}a\left(\theta \right)}.$$

The angles corresponding to the *Q* largest peaks are the directions of the sources.

## 3. The Direct Coarray Interpolation Approach for Direction Finding

For several sparse array structures (e.g., coprime array), not all the lags are consecutive. This means that the lags out of consecutive range cannot be used to perform coarray-based MUSIC, thus decreasing the number of DOFs. An interpolation method via nuclear norm minimization has been proposed in [18] to fill the holes. As such, the lags out of consecutive range can be utilized. However, the noise item $\tilde{E}$ is not suppressed in [18]. In addition, the structure of the covariance matrix of ULA is not taken into account, which will increase the computational complexity. It is well known that the covariance matrix of a ULA is Hermitian and Toeplitz. Thus, we can exactly know the entire matrix, even if some rows or columns are missing. For instance, consider a ULA with locations {0,1,2,3}. Then, the covariance matrix is

(16) $$R_{\mathrm{ULA}}=\left[\begin{array}{cccc} E[x_{1}x_{1}^{*}] & E[x_{1}x_{2}^{*}] & E[x_{1}x_{3}^{*}] & E[x_{1}x_{4}^{*}]\\ E[x_{2}x_{1}^{*}] & E[x_{2}x_{2}^{*}] & E[x_{2}x_{3}^{*}] & E[x_{2}x_{4}^{*}]\\ E[x_{3}x_{1}^{*}] & E[x_{3}x_{2}^{*}] & E[x_{3}x_{3}^{*}] & E[x_{3}x_{4}^{*}]\\ E[x_{4}x_{1}^{*}] & E[x_{4}x_{2}^{*}] & E[x_{4}x_{3}^{*}] & E[x_{4}x_{4}^{*}] \end{array}\right],$$

which is a Hermitian and Teoplitz matrix. If we remove the sensor with location 2, the 3rd row and the 3rd column are missed. However, we can still recover $R_{\mathrm{ULA}}$ by utilizing other existing entries. To be specific, $E[x_{1}x_{3}^{*}]$ equals $E[x_{2}x_{4}^{*}]$, thus making it possible to fill $E[x_{1}x_{3}^{*}]$ with $E[x_{2}x_{4}^{*}]$. Similarly, $R_{\mathrm{ULA}}$ can be fully recovered. Overall, the number of variances of an L×L Hermitian and Toeplitz matrix is *L*. So, the complexity is significantly reduced if the matrix structure is considered. Inspired by this, a denoising operation is added to improve the DOA estimation accuracy in [19]. Although the array structure is utilized in the denoising operation, the algorithm in [19] still suffers high computational burden because two convex problems are optimized. In addition, for the algorithms in [18,19], the reshaping operation (11) is still required to select the data vector of coarray, thus making the interpolation process complex.

According to (16), $R_{V}$ can be represented by a vector $u=\left[u_{1},\ldots ,u_{d_{L}+1}\right]$ as

(17) $$R_{V}=T\left(u\right)=\left[\begin{array}{cccc} u_{1} & u_{2} & \ldots & u_{d_{L}+1}\\ u_{2}^{*} & u_{1} & \ldots & u_{d_{L}}\\ \vdots & \vdots & \ddots & \vdots \\ u_{d_{L}+1}^{*} & u_{d_{L}}^{*} & \ldots & u_{1} \end{array}\right].$$

If the number of sources is less than the cardinality of V (i.e., $Q\leq d_{L}+1$), $R_{V}^{\left(sig\right)}=A_{V}R_{ss}A_{V}^{H}$ is low-rank (rank *Q*). Note that this does not mean the number of degrees of freedom (DOFs) is up to $d_{L}$ by using $R_{V}$ to perform MUSIC. The number of DOFs still depends on the unique lags that are contained in coarray, because no additional information about the DOAs is induced after interpolating the holes. For coprime arrays, as defined in (1), the number of unique lags is 3MN+M−N (Lemma 1 in [18]).

In this section, we propose a novel approach to recover $R_{V}$ directly from ${\tilde{R}}_{S}$. The selection of unique entries in ${\tilde{R}}_{S}$ is no longer required in the proposed approach. In addition, the denoising operation and coarray interpolation are performed simultaneously, thus significantly reducing the computational complexity and suppressing the noise. According to (6), many entries in $R_{V}^{\left(sig\right)}$ are equal to the entries in $R_{S}$ in a noise-free case. Thus, the recovery of $R_{V}^{\left(sig\right)}$ is a typical matrix completion problem [23,24]. The low rank matrix $R_{V}^{\left(sig\right)}$ can be recovered from $O\left[\left(d_{L}+1\right)Q\mathrm{polylog}\left(d_{L}+1\right)\right]$ measurements about $R_{V}^{\left(sig\right)}$ by minimizing the rank of $R_{V}^{\left(sig\right)}$[24]. However, the rank function is not a convex function, thus making the rank minimization problem difficult to solve. The nuclear norm, defined as the sum of the singular values, is a convex relaxation of the rank function. Then, we propose the following nuclear norm minimization problem to directly recover the entire $R_{V}^{\left(sig\right)}=T\left(u^{\circ }\right)$ from ${\tilde{R}}_{S}$.

(18) $$\begin{array}{cc} \underset{u^{\circ }}{\mathrm{minimize}}\; & ∥T\left(u^{\circ }\right){∥}_{*}\\ \mathrm{subject}\;\mathrm{to}\; & ∥Q^{T}T\left(u^{\circ }\right)Q-{\tilde{R}}_{S}{∥}_{F}\leq \epsilon \\ & T(u^{\circ })⪰0, \end{array}$$

where ${∥\cdot ∥}_{*}$ and ${∥\cdot ∥}_{F}$ respectively represent the nuclear norm and the Frobenius norm, and ϵ depends on the noise level. Since the sample covariance matrix ${\tilde{R}}_{S}$ is contaminated by noise, the first constraint $∥Q^{T}T\left(u^{\circ }\right)Q-{\tilde{R}}_{S}{∥}_{F}\leq \epsilon$ aims to suppress the noise contained in ${\tilde{R}}_{S}$. $T(u^{\circ })⪰0$ means that T(u) is a positive semidefinite matrix, which is the property of covariance matrix. By optimizing (18), we can directly obtain $R_{V}^{\left(sig\right)}$ without the reshaping operation as $R_{V}^{\left(sig\right)}=T\left(u^{\circ }\right)$. In addition, the rank of $R_{V}^{\left(sig\right)}$ is already recovered. Thus, subspace-based DOA estimation algorithms can be readily performed by using $R_{V}^{\left(sig\right)}$. It is worth noting that the proposed approach can be used in any structures of sparse arrays as long as the number of missing entries in ${\tilde{R}}_{S}$ is not too large. After obtaining the optimal solution to (18), we can use MUSIC to estimate the DOAs.

We then compare the proposed approach with the coarray interpolation in [18] and the hybrid approach in [19] and show the main advantages of the proposed approach.

1. The hybrid approach requires two convex optimization problems to get the denoised covariance matrix, while the proposed approach only needs to solve one convex optimization problem. Thus, the computational complexity is significantly reduced.
2. In the hybrid approach, after obtaining the covariance matrix of the received signal, we should first vectorize the covariance matrix and then select the entries corresponding to the unique lags in coarray. Only after these preprocessings can we further interpolate the holes and suppress the noise. However, in the proposed approach, the interpolated and denoised covariance matrix of coarray can be obtained directly from the covariance matrix of the received signal, thus significantly simplifying the processing.
3. Compared with the coarray interpolation, the denoising operation is added by utilizing the low rank property of covariance matrix of coarray. Thus, the estimation accuracy is better than the coarray interpolation. As indicated by the numerical simulations, the performance is similar to—sometimes even better than—the hybrid approach.
4. Finally, an interesting result indicated by the simulation results is that the angular resolution of the proposed approach is better than the hybrid approach and the coarray interpolation.

The entire process of the proposed approach is summarized in Table 1.

## 4. Simulation Results

In the following simulations, a coprime array with M=3 and N=5 (namely, S={0,3,5,6,9,10,12,15,20,25}) is used. The corresponding difference coarray is {0,±1,…,±17,±19,±20,±22,±25}. We first intuitively examine the performance of direct coarray interpolation by showing the MUSIC spectrum. Then, the estimation performance of the proposed approach is examined by calculating the root mean square error (RMSE) versus input signal-to-noise ratio (SNR) and the number of snapshots. I=500 independent Monte Carlo trials are repeated in each simulation. The performances of the coarray interpolation approach [18] and the hybrid approach [19] are simulated for comparison. The definition of RMSE is

$$\mathrm{RMSE}=\sqrt{\frac{1}{IQ}\sum_{i=1}^{I}\sum_{q=1}^{Q}{\left({\tilde{\theta }}_{q}\left(i\right)-\theta_{q}\right)}^{2}},$$

where ${\tilde{\theta }}_{q}\left(i\right)$ is the estimate of $\theta_{q}$ for the *i*th Monte Carlo trial, i=1,…,I. Finally, the angular resolution in the two close sources scenario is examined.

### 4.1. MUSIC Spectrum

**Figure 1:**
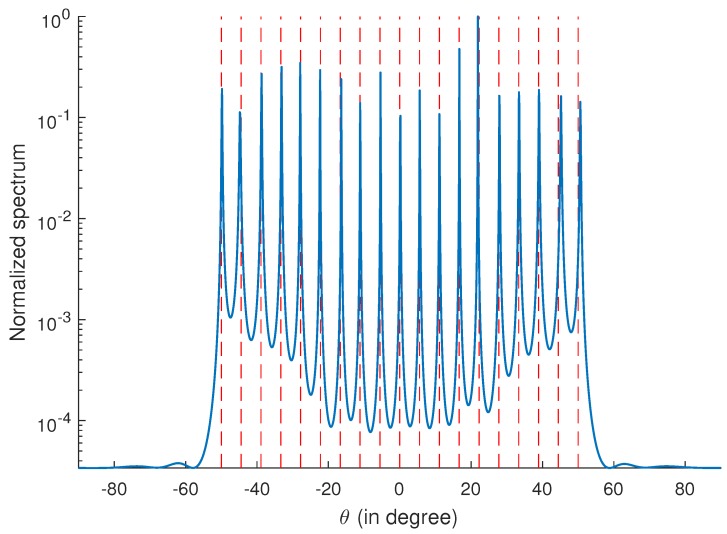
DOA estimation using the proposed algorithm. Red dashed lines are the true DOAs. Q=19 sources distributed uniformly from $\left[-50^{\circ },50^{\circ }\right]$ impinge on the coprime array. The signal-to-noise ratio (SNR) is 0 dB, the number of snapshots is 500, and ϵ is set as 8.

The consecutive range of the coarray is from −17 to 17, indicating that the resolvable number of sources is 17 without filling the holes. In this subsection, we consider 19 uncorrelated sources which are uniformly distributed in $\left[-50^{\circ },50^{\circ }\right]$. A SNR of 0 dB and 500 snapshots are used, and ϵ is set as 8. The spatial spectrum is depicted in Figure 1. It is obvious that the directions are correctly estimated, which verifies that the holes in the coarray are filled successfully.

### 4.2. Estimation Performance

**Figure 2:**
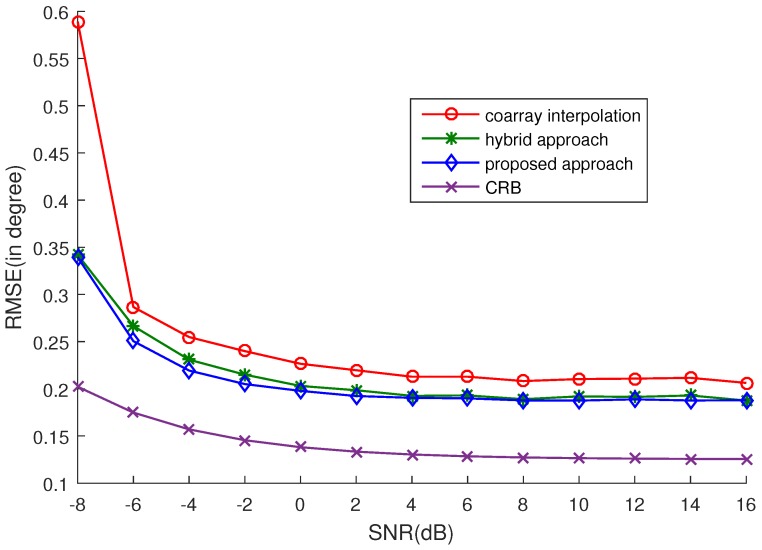
RMSE vs. SNR. Five hundred Monte Carlo trials are conducted and Q=16 sources uniformly distributed in $\left[-50^{\circ },50^{\circ }\right]$ are considered. The number of snapshots is K=500 and the selection of ϵ is listed in Table 2.

**Figure 3:**
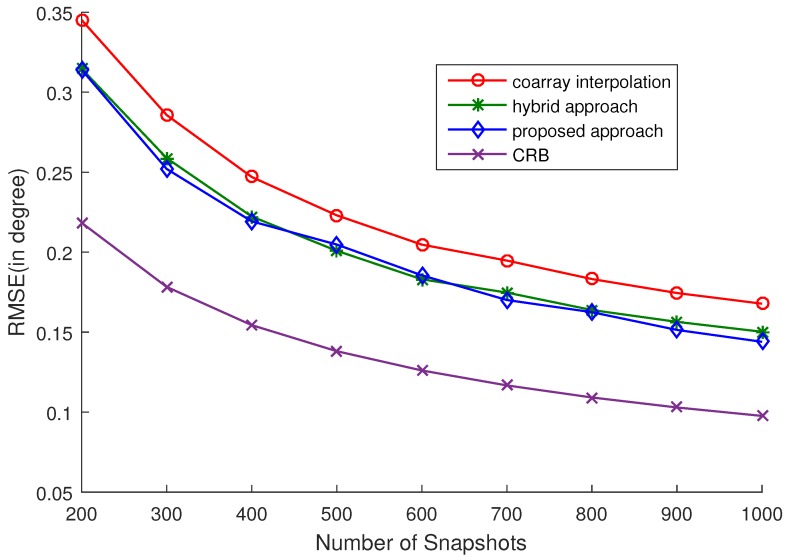
RMSE vs. the number of snapshots. Five hundred Monte Carlo trials are conducted and Q=16 sources uniformly distributed in $\left[-50^{\circ },50^{\circ }\right]$ are considered. The SNR is 0 dB and the selection of ϵ is listed in Table 3.

In this subsection, the RMSEs versus SNR and the number of snapshots are computed to further exploit the estimation performance of the proposed approach. 16 uncorrelated sources are considered. The RMSEs versus SNR and the number of snapshots are plotted in Figure 2 and Figure 3, respectively. Five hundred snapshots are used in Figure 2, and 0 dB SNR is set in Figure 3. The selection of ϵ is listed in Table 2 and Table 3, corresponding to Figure 2 and Figure 3, respectively. We can easily see that the proposed approach outperforms the other two approaches from the simulations. Although the hybrid approach can achieve a similar accuracy to the proposed approach, the computational complexity of the proposed approach is much lower than that of the hybrid approach. The reason is that two nuclear norm minimization problems need to be optimized in the hybrid approach, while only one nuclear norm minimization problem is optimized in the proposed approach. Experimentally, under the same conditions, it takes 102.0889 s to run 50 trials for the hybrid approach and 75.5295 s for the proposed approach. Note that as analyzed in [16], the estimation accuracy of coarray-based MUSIC cannot attain the CRB when there are more sources than physical sensors. So, there is a gap between the RMSE and the CRB.

### 4.3. Resolution Capability

**Figure 4:**
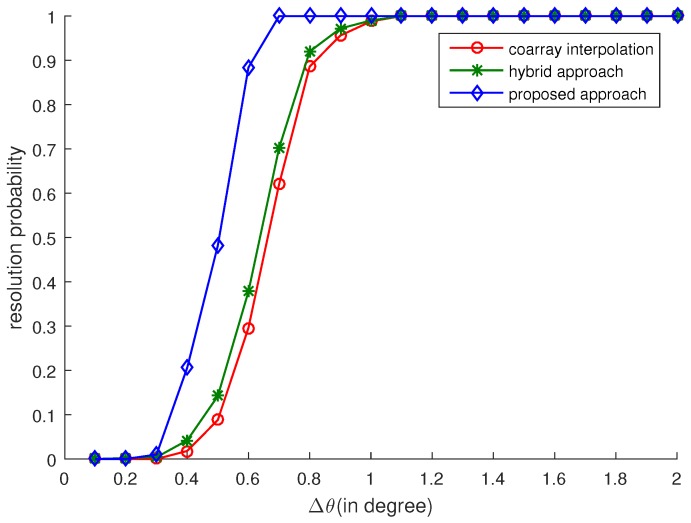
Resolution probability vs. Δθ. Two uncorrelated sources are located at $30^{\circ }\pm \Delta \theta$. SNR is 0 dB and the number of snapshots is 200.

The angular resolution capability is examined in this subsection. Two sources are located in the directions $\theta_{1}=30^{\circ }-\Delta \theta$ and $\theta_{2}=30^{\circ }+\Delta \theta$. We define that the two sources are correctly resolved when there are two peaks in the MUSIC spectrum and the estimated DOAs ${\hat{\theta }}_{1}$ and ${\hat{\theta }}_{2}$ satisfies $|{\hat{\theta }}_{1}-\theta_{1}|<\Delta \theta$ and $|{\hat{\theta }}_{2}-\theta_{2}|<\Delta \theta$. The SNR is 0 dB and the number of snapshots is 200. Δθ varies from 0.1 to 2 degrees and the ϵ is set as 4. Five hundred Monte-Carlo trials are conducted for each Δθ to acquire the resolution probability. The resolution probability versus Δθ is shown in Figure 4. We find that the proposed approach can achieve a higher angular resolution than both the coarray interpolation and the hybrid approach.

## 5. Conclusions

A novel DOA estimation approach was proposed in this paper to achieve accurate DOA estimation with a low complexity. The main contribution of this paper is the nuclear norm minimization problem. As a result, the reshaping and spatial smoothing operations are not required. In addition, the noise effect is suppressed by optimizing the nuclear norm minimization problem. Furthermore, the angular resolution is also improved by using the proposed approach. Simulation results validated the effectiveness of the proposed approach.

## Figures and Tables

**Table 1:** Direct approach for coarray interpolation. DOA: direction of arrival.

**Input**	The received signal vector x(t) with time index t=1,2,…,T
**Output**	DOA Estimation
**Step 1**	Compute the covariance matrix ${\tilde{R}}_{S}=\sum_{t=1}^{T}{\tilde{x}}_{S}\left(t\right){\tilde{x}}_{S}^{H}\left(t\right)$.
**Step 2**	Optimize (18) to obtain $R_{V}^{☆}$ as $R_{V}^{☆}=T\left(u^{☆}\right)$.
**Step 3**	Perform eigen-decomposition of $R_{V}^{☆}$ and obtain the corresponding noise subspace $U_{N}$.
**Step 4**	Compute (15) and the *Q* largest solutions are the estimation of DOAs.

**Table 2:** ϵ with respect to different numbers of snapshots.

snapshots	200	300	…	900	1000
ϵ	12	11.5	…	8.5	8

**Table 3:** ϵ with respect to different SNRs.

SNR (dB)	−8	−6	−4	−2	0	2	…	16
ϵ	15	14	13	12	10	10	…	10
